# Supplementary material for: Are carbon emissions trading and green financial instruments synergistic? -Comprehensive quantitative research based on content analysis
Source: PLoS One. 2024 Mar 7;19(3):e0298601. doi: 10.1371/journal.pone.0298601 (PMC10919601; doi:10.1371/journal.pone.0298601)
Supplement: S1 Data — (ZIP) [file pone.0298601.s001.zip › Data/地级市Z_F工作报告.zip!/╣π╢1⁄2/╔╪╣╪╩╨/└┤╫╘╣1⁄2╓┌║┼í╛┬φ┐╦╩2╛▌═°í┐_2014.docx]

各位代表：

　　我代表市人民政府向大会作工作报告，请予审议，并请政协各位委员和其他列席人员提出意见。

　　2013年工作回顾

　　2013年，在省委、省政府和市委的正确领导下，在市人大及其常委会和市政协的监督支持下，市政府积极抢抓省进一步促进粤东西北地区振兴发展的重大机遇，全力推动经济社会加快发展，全面实现市十三届人大三次会议确定的主要预期目标。

　　经济迈上新台阶。主要经济指标增幅高于全省平均水平。全市生产总值突破1000亿元、达1010亿元，增长12.1%；人均生产总值3.5万元，增长11.3%。来源于韶关的财政总收入突破200亿元、达244.9亿元，增长33.9%；地方公共财政预算收入71.7亿元，增长16.5%。产业转型稳步推进。农业增产增收，工业较快发展，服务业稳步提升。东莞大岭山（南雄）产业园成功扩园。我市连续6年通过国家科技进步考核，连续2届被评为全国科技进步考核先进市，连续3年获得国家产学研结合工作奖，2013年获得国家技术发明二等奖，我市科技获奖创历史最好成绩。东阳光铝业公司技术中心被确认为国家级技术中心。交通银行、东亚银行在韶首家分支机构开业，汇丰银行新增一家支行。基础设施和重点项目建设加快。固定资产投资增长21.2%。乐昌峡水利枢纽工程、韶能生物质能发电项目并网发电，韶赣铁路和广乐、大广高速公路加快建设，韶关电厂“上大压小”、新丰稀土分离项目获国家核准批复。县域经济和民营经济持续较快发展，占全市经济比重均提高到51.7%。

　　城乡建设展现新面貌。中心城市扩容提质有序开展。城建重点工程进展顺利，项目数量和投资额均创历史新高。芙蓉新区及其管委会获准成立，芙蓉新区发展总体规划获省政府批准。赤水安置区建成，芙蓉、西联安置区启动建设。43号路建成通车，道路建设工作全面铺开。武警作战指挥中心建成并投入使用。韶关实验中学、联通、恒大、碧桂园、保利等项目建设加快。老城区四大出口与工业西片区内涝整治等市政项目以及金色江湾、万通城等一批“三旧”改造项目加快推进。全市依法拆除违法建筑面积20.7万平方米。新农村建设扎实推进。完成通自然村公路路面硬化582公里。新一轮扶贫开发开局良好。生态文明建设成效显著。我市成为国家节能减排财政政策综合示范城市。封山育林1200万亩，春季造林42.9万亩。丹霞山被列为国家生态旅游示范区。始兴县成为全省首批农村环境保护连片整治试点。市区更新投放89辆CNG出租车、50辆纯电动出租车。淘汰落后水泥产能248万吨、电池17.5万千伏安时。完成省下达的节能减排任务。

　　民生福祉得到新改善。人民生活水平不断提高。全市城镇居民人均可支配收入、农村居民人均纯收入、城乡居民储蓄存款余额分别增长10.4%、11.7%和12.3%。居民消费价格指数涨幅低于全国、全省平均水平。市政府公开承诺办好的10件民生实事当年投入3.2亿元，其中5件实事已经完成，另外5件实事因投资和征地拆迁量较大、需跨年实施，正按计划推进；市政府各部门（单位）承诺的54件实事基本完成。农村劳动力技能培训转移就业工作连续5年进入全省优秀行列。城镇登记失业率为2.35%。落实底线民生保障各项政策，社保、低保、五保待遇提高，住房保障力度加大。有效应对3次严重洪涝灾害，灾后复产重建家园工作全面完成。社会事业全面发展。完成教育创强工作。基本解决符合条件的异地务工人员子女完成义务教育后参加初中升学考试问题。市特殊教育学校完成搬迁新建并投入使用。省级高技能公共实训基地晋升为国家级。基层卫生医疗机构全面实行基本药物制度，县级公立医院改革试点、平价医疗服务工作有序推进。粤北医院门急诊医技综合楼、市铁路医院中华健康快车白内障治疗中心、市残疾人康复中心以及4个县级医院业务大楼建成使用。完成国家和省文化惠民工程任务。国家卫生城市复审通过，全国文明城市创建有序推进。建成22个乡镇农民健身工程。成功举办世界张氏总会第六届恳亲大会、纪念六祖惠能圆寂1300周年暨2013广东禅宗六祖文化节（韶关）系列活动。乳源“瑶族十月朝”被评为全省群众性文化活动优秀品牌。社会治理不断加强。基层综合服务管理平台进一步健全。社工人才试点和购买社工服务试点工作进展顺利。安全生产总体平稳，完成省下达的控制目标。平安韶关创建和“两建”工作取得新进展。

　　政府建设取得新成效。注重发挥民主党派、工商联、无党派人士和人民团体的作用，认真办理人大代表建议、政协提案，深入推进依法行政。政府职能转变和机构改革工作扎实推进。改革完善市县食品药品监督管理体制，推进市县卫生和计生部门职能转变。精减市直事业单位154个，精减率达45.4%。市级取消行政审批事项26项、下放23项、转移4项、委托2项。市县两级网上办事大厅和电子政务办公业务系统建成运行。94个乡镇建成便民服务中心。落实中央“八项规定”，着力纠正“四风”。市级考核项目大幅减少。政务公开、行政监察、审计监督、网络问政、纠风工作力度加大，严肃查处了一批违法违纪案件。

　　各位代表，上述成绩来之不易，这是全市人民团结奋斗的结果。在此，我代表市人民政府，向全市人民，向驻韶部队官兵，向各民主党派、人民团体、社会各界人士，向关心、支持韶关发展的港澳台同胞和海内外朋友，表示衷心的感谢！

　　我们也清醒认识到存在的问题。主要是：经济总量小，人均水平低；产业支撑力不强，创新能力弱；重大项目和骨干企业少，工业投资增长不快；城镇化步伐还不够大，中心城区辐射带动力不强；资源环境承载压力加大，节能减排任务繁重；部门单位服务水平和办事效率与人民群众的期望还有差距，政务环境需要进一步优化。对此，我们将采取更有力的措施积极加以解决。

　　2014年工作安排

　　2014年政府工作的总体要求是：坚持发展这个硬道理，认真落实党的十八大和十八届二中、三中全会精神及省委、省政府、市委的决策部署，坚持稳中求进，以深化改革为统领，以扩大开放为动力，以加快新型工业化和城镇化为突破口，奋力开创绿色转型、振兴发展新局面。主要预期目标是：生产总值增长11%以上；人均生产总值增长10%；地方公共财政预算收入增长11%以上；固定资产投资增长18%；社会消费品零售总额增长14%；城镇居民人均可支配收入增长9%，农村居民人均纯收入增长10%；居民消费价格指数涨幅控制在3.5%左右；城镇登记失业率控制在3%以内；完成省下达的节能减排等各项任务。

　　围绕上述目标，今年突出抓好六个方面的工作：

　　一、加强经济建设

　　继续抓住经济建设这个中心和转变经济发展方式这条主线，积极破解难题，巩固和发展经济稳中向好的势头，并加强区域合作，与北江流域相关地区共同努力，打造北江经济带。

　　明确重点，突出三大抓手。着力扩大投资，优化资金投向，强化重大发展平台，增强经济发展动力。一要大力推进基础设施提档升级。推动韶赣铁路、广乐高速公路建成通车。重点推进大广、武深、汕昆高速公路和武广高铁乐昌东站、北江航道整治、韶关机场扩建等重大交通基础设施项目，以及南水水库供水工程、韶关电厂“上大压小”、国电粤华煤矸石发电、始兴羊角山风力发电、市区第四和第五污水处理厂、智慧城市等重大民生、能源、环保、市政、信息化基础设施建设。创新基础设施建设方式，探索引入社会资本加快湖南莽山黄沙至乐昌长来、韶关至新丰高速公路新江至六里段等高速公路项目建设。二要大力促进产业园区扩能增效。完成莞韶产业园扩园工作，积极推进北扩，加快打造“广东装备制造之都”。推进始兴产业园扩园，加快仁化、翁源、新丰、乳源产业园区的申报，实现省级产业园县域全覆盖，促进县域经济加快发展。赋予莞韶产业园市级经济社会管理权限，各县（市、区）相应扩大园区管理权限。抓住东莞对口帮扶的重大机遇，按“一园一城七组团”的思路，优化我市产业园布局和发展路径。进一步明确园区产业定位，着力引进优质项目，拓展延伸产业链，培育产业集群。加大园区基础设施、生活配套和公共服务平台建设投入，提高园区产业承接能力和竞争力。有序和谐开展土地征收，拓展园区发展空间。探索建立利益共享机制，加大合作共建力度，发挥好东韶投资开发公司作用。三要大力推进城市扩容提质。坚持以人为本，抓住农业转移人口市民化这个核心，提升城镇建设水平和服务功能，加快城乡一体化发展；注重生态保护和文化传承；健全新型城镇化体制机制，拓宽城市建设投融资渠道，鼓励社会资本参与；强化规划引领作用，着力推进经济社会、城乡建设、土地利用等规划衔接整合工作。加快推进芙蓉新区起步区城市建设。扎实推进农民安置房建设和旧村拆迁。铺开芙蓉新城市政设施、公共服务设施和水系建设。加快南华路、滨江路（一期）等18条道路以及新白线、芙蓉隧道建设，加强公交服务。完成韶关实验中学一期并推进二期建设，加快医院、韶关联通、移动服务大楼、农贸市场等建设。加快金融服务区、中央商务区、粤北亚太财富中心和恒大、碧桂园五星级酒店等项目建设，鼓励有条件的企业到新城建设总部大楼，促进新城服务产业聚集。推动碧桂园、恒大、保利、信德等地产项目在绿化、停车位等方面提供更好的人居环境，逐步营造芙蓉新城明显优于老城区的城市功能。推进老城区扩容提质。继续推进市区四大出口、东环线、上饶路、移山路、群康路建设。建设跨武江、浈江步行桥。加快工业西路等城市内涝多发区整治。完善“三旧”改造政策，加强规划引导和监管。推进棚户区和城中村改造。加强市区交通拥堵整治。推进城市网格化管理，开展户外广告、占道经营等专项整治。提高县城和中心镇的人口和产业集聚度。加强旅游城镇规划建设，完成城镇体系规划和村镇体系规划，分类指导乡镇建设，加强规模较大中心镇建设。各县（市）要结合当地实际，确定建设一个新区或者集中连片改造一个老城区，加快城镇化进程。

　　夯实基础，推动产业转型升级。坚持做大产业规模与提升产业水平互动并进，抓住重大项目、重大平台、重点企业、重大科技专项和信息化，促进产业提质增效升级，并做好我市全国第三次经济普查工作。一要加快新型工业化步伐。促进各类工业企业协同稳定发展。加强大型骨干企业培育，实行“一企一策”，着力培育一批主营业务收入超10亿元、50亿元、100亿元的企业，带动产业链延伸发展。落实扶持中小微企业政策措施。鼓励扶持个体户发展为企业、规下企业发展为规上企业。推进产业结构调整和产业升级。用好资源枯竭型城市可持续发展政策，优化发展资源型产业，着力提高装备制造、精细化工、氟化工、生物医药、新型建材、新材料等接续替代产业的工业占比。支持企业技术改造、加强管理和科技创新，向产业价值链的高端迈进。以管资本为主加强国有资产监管。支持企业增资扩产和兼并重组，鼓励国内外行业优势企业收购兼并我市工业企业。推进老工业基地搬迁改造，鼓励引导老城区工业企业向产业园转移。推进小水电增效扩容改造。实施质量强市、技术标准和品牌带动战略，落实企业研发费用税前扣除和高新技术企业税收优惠，进一步优化创新环境。二要加快发展现代服务业。提升壮大旅游产业。加快环丹霞山生态旅游产业园、南雄奥威斯乐园、曲江大森林温泉世界度假村、新丰云髻山景区等旅游重大项目建设，打造国家旅游产业集聚区和南岭生态旅游集散地。深化旅游体制机制改革，办好旅游宣传推介主题活动，推出一批团队、散客等旅游服务套餐和特色纪念品。加强城市整体形象营销和对外合作，发展乡村旅游和农家乐、瑶家乐。加强旅游基础建设，促进旅游和其他行业融合发展。加快发展金融业。鼓励银行业加大信贷投放，争取贷存比提高2个百分点。推进农信社改制和地方金融机构扩量提质。积极吸引金融机构落户。推动农村金融创新，抓好“政银保”项目，发展政策性涉农保险，争取成为省农村金融改革试点市。壮大商贸流通业。以抓好市区东环线商贸物流园区建设为重点，以美吉特、新雪域等项目为带动，积极引导各类专业市场集聚发展。推进粤北国际物流园、中烟韶关物流基地、粤北金属物流交易中心、龙归物流园、南雄精细化工交易中心、新丰横江商贸物流城、韶关义乌国际小商品商贸城等项目建设。加强现代物流配送和专业物流中心建设。发展总部经济，大力推动重点企业原材料采购、产品销售业务本地化。积极发展电子商务和信息服务业。以莞韶城电子商务与信息服务项目为重点，引进优势电商企业，培育本地电商龙头企业，完善面向中小企业的电子商务服务。以“粤北硅谷”项目为重点，着力引进一批信息服务企业。重视发展健康服务业。加大支持引导力度，着力培育发展中医保健、养生养老、健康体检、体育健身等健康服务产业。此外，还要规范发展物业管理、快递业等服务业。三要着力发展山区特色效益农业，促进新农村建设。推进适度规模经营，鼓励农村土地承包经营权向专业大户、家庭农场、农民合作社、农业企业流转，培育新型经营主体。提升农业产业化水平，支持做大农产品流通加工业，加快农业科技创新推广。优化农业发展载体，重点加快仁化国家现代农业示范区、粤北现代农业示范园区、粤台农业合作试验区建设。提升农业生产能力和防灾抗灾能力，推进高标准基本农田建设，强化粮食安全保障能力。开展中小河流治理、小流域综合整治、中小型灌区改造、病险水库除险加固。大力发展林业产业，壮大乡土珍贵阔叶树、油茶、竹子、花卉苗木等特色产业，大力发展茶叶、铁皮石斛、中草药等林下经济和家具制造业，拓展林业经济增长点。探索农村建设新模式，创新城乡建设用地增减挂钩政策实施机制，使之与旧村庄改造有机结合，积极引导社会资本参与扶贫开发和幸福村居创建，加强农村危旧房改造，推进“两不具备”村庄搬迁改造。稳妥推进农村土地承包经营权、宅基地的确权登记和建库工作。做好新一轮扶贫开发“双到”工作，加快革命老区、民族地区和水库移民区发展。

　　加强对经济工作的协调，着力破解瓶颈难题。加强项目管理，服务项目建设。深入实施“百项工程兴韶关”活动，加强重点项目调度，建立健全市领导挂点联系和项目各方联动工作机制，开展重点项目互学互评活动，严格督促问责。制订重点项目推进时间表，加强协调服务，对企业投资项目推行网上在线备案，力争更多项目获批、落地和开工。建立重大项目审批绿色通道制度，实行并联审批加承诺制的审批模式。加强土地资源统筹配置，完善土地、房屋征收政策和工作机制。重点推进韶钢技改提升、新丰稀土分离等21项现代产业工程建设。积极推进华电南雄热电冷联供、正菱集团专用汽车制造等前期预备项目，完善重大项目储备库。加强招商引资工作，提高开放水平。明确招商思路，制定实施产业链招商行动计划。开展招商引资年活动，实施招商引资“一把手”工程，加强中介招商、以商引商和莞韶联合招商，注重质量招商，着力招大引强，力争引进一批10亿元或1亿美元以上的产业项目，狠抓项目跟踪落实。完成省下达的外资外贸工作任务。加强财税金融工作，强化资金保障。强化财税运行分析和税收征管，整合各类财政扶持政策资金，提高财政资金使用绩效，集中财力办大事。完善助保贷机制。加强融资平台建设，支持企业改制上市、发行债券，努力缓解中小微企业融资困难。完善征信体系，优化信息共享、有效担保、登记公证等金融发展环境。

　　二、加强文化建设

　　实施“文化名城”战略，建立健全现代公共文化服务体系和现代文化市场体系。

　　加强城市主题文化建设。制定相关规划，加强城市主题文化设施及主题文化产业项目建设。加强文明城乡建设。倡导培育和践行社会主义核心价值观，推进全国文明城市创建工作，强化公民道德建设和未成年人思想道德建设，着力开展以道德讲堂、志愿服务为重点的全民修身活动，以文明交通、文明餐桌、文明上网为重点的文明引导行动。加强文化惠民工程建设。深化图书馆、博物馆、文化馆等文化事业单位改革。加快市新图书馆、新文化馆、新档案馆建设，完成县镇村三级文化设施达标年度任务。推动公共文化服务向社区、企业和农村延伸。抓好农村电影放映工程。加快实施县镇数字影院全覆盖工程。积极落实省广播电视节目无线覆盖工程。扶持促进民间文化艺术发展。加强文化遗产和文物的保护。加强历史文化街区、历史文化名村、古村落和少数民族特色村寨的规划保护利用。推进广富新街历史文化街区维修改造。加强文化产业建设。出台政府购买公共文化服务目录及实施方案，引导社会资本投资文化产业。支持小微文化企业发展，推进国有经营性文化单位转企改制，着力培育骨干文化企业。推进大南华文化创意产业园建设。规范文化市场秩序。

　　三、加强社会建设

　　以增进民生福祉、促进社会公平正义为出发点和落脚点，强化公共服务和社会管理职能。

　　更好保障和改善民生。健全工资支付保障制度。加强就业创业服务。促进以高校毕业生为重点的青年就业和农村转移劳动力、城镇困难人员、退役军人就业。推进社保扩面，提高社保参保率、保险待遇和经办服务水平。充分发挥住房公积金对改善居民居住条件的作用。完善保障性住房建设、分配、管理和退出机制，新开工建设保障房3341套。建设与田螺冲棚改工程配套的学校、医院、垃圾中转站、公交站等公建设施。强化社会救助，推进城乡低保、五保供养制度与城乡居民社会养老保险制度配套衔接，发展社会福利和慈善事业，加强养老、恤孤、助残工作。

　　提升公共服务水平。加强义务教育标准化学校建设，推进县域内城乡义务教育均衡发展。整合提质市区教育资源。加强流动人口子女教育管理。加强高中教育、学前教育和特殊教育发展。支持韶关学院提升办学层次和迎接教育部审核性评估。深化医药卫生体制改革。推进县级公立医院改革和平价医疗服务工作。完善市县镇村四级公共卫生服务体系。强化卫生应急、疾病预防控制、妇幼保健和卫生监督工作。建设粤北医院全科医生临床培训大楼。构建城乡一体化公共交通服务体系。进一步提高人口计生服务水平。建成20个乡镇农民体育健身广场，深入开展全民健身活动。推进机关事业单位的体育设施向公众开放。办好市十四届运动会，备战2014年省青少年锦标赛和第五届省少数民族传统体育运动会。

　　加强和创新社会治理。加强社会信用体系和市场监管体系建设。落实领导接访制度，改革涉法涉诉信访工作，加强社会风险排查化解，提升流动人口动态服务管理效能，深化社会治安综合治理，深入创建平安韶关。推进户籍制度改革，有序推进农业转移人口市民化。创新劳动关系协调机制，构建和谐劳动关系。建立健全重大决策社会稳定风险评估制度。建立健全食品药品安全监管网络。创新安全生产管理模式，落实企业主体责任，强化安全生产防控体系建设，严格事故责任追究。健全打击“违法用地、违法建筑”联动机制，坚决打击“两违”行为。推进县（市、区）应急平台建设，提高应急管理能力。加大网络社会管理，及时回应网民关切。改进住宅小区物业管理，加强业主委员会建设，健全基层综合服务管理平台，深化城乡社区建设。建立社会组织孵化基地，规范发展社会组织。争创第十届全国、全省双拥模范城。

　　今年，市政府将在抓好以往承诺民生实事的同时，办好8件实事：1.提高底线民生保障水平；2.提高城乡居民医保补助标准，每人年补助标准由280元提高到320元；3.提高人均基本公共卫生服务经费标准，由30元提高到35元以上；4.开展政策性水稻种植保险；5.资助市公交公司增购100辆LNG新能源公交车；6.建设市社会福利院老年人综合服务大楼；7.建设一批小公园、小广场、小运动场、小停车场、小菜市场等“五小”惠民项目；8.推进市区饮用水取水点调整优化工程和武江北路、靖村排水渠截污工程。

　　四、加强生态文明建设

　　实施主体功能区制度，划定并公布实施我市生态控制线，加强生态建设和环境保护，推动绿色、低碳、循环发展。

　　提升生态环境质量。积极推进“广东绿色生态第一市”建设。加强封山育林工作，加快生态景观林带、森林碳汇、森林进城围城、乡村绿化美化等重点林业工程和绿道网建设。加强自然遗产地、重要水源涵养区、石漠化地区、水土流失区、生物多样性保护区、地质灾害和荒废矿区等区域的生态保护与综合治理。新增30个森林公园和1个湿地公园。加快航空护林基地和森林远程视频监控系统建设。努力营建以乡土阔叶树种为主的混交林，提高森林质量。健全林木采伐与更新“一本帐”的管理机制。

　　提高环境保护水平。落实省政府“十二五”后半期节能行动计划、主要污染物总量减排行动计划、大气污染防治方案，加强节能减排工作。强化节能减排目标责任和体制机制创新，强化财政资金引导作用，以奖代补、以奖促治。启动电机能效提升计划。发展绿色节能建筑，完成市区路灯LED改造。加快污水处理设施与截污管网、城乡污泥处置和规模化养殖场污染治理等节能减排项目建设。加快淘汰“黄标车”。推进韶冶整体搬迁升级改造。提高环境监管和应急处置水平。在市区开展PM2.5等项目监测工作。加强重点区域流域空气、水体、土壤污染综合治理，搞好跨省、市界水质断面污染监控。合理划定饮用水源保护区，加强饮用水源保护。加强农业面源污染防治，综合整治城乡环境。

　　节约集约利用资源。落实最严格的水资源管理制度以及供电与产出挂钩、差别水价、企业用地与产出挂钩等政策。加强批而未供、供而未用土地消化和低丘缓坡地综合开发利用，统筹用好建设用地地上地下空间。整顿规范矿产资源开发秩序，提高新矿权设置门槛，整合现有矿权，争取设置有利我市发展的矿权。落实生态产业发展规划，推进粤北危险废物处理处置中心、市再生资源集散交易中心建设，大力发展循环经济，提高资源回收利用水平，推进生态资源产业化。建立矿山企业生态修复保证金制度。

　　五、加强政府建设

　　市政府将牢记宗旨，努力为民、公正、依法、廉洁用权。

　　严格依法行政。自觉接受监督。认真落实人大及其常委会的决议决定，认真办理人大代表建议和政协委员提案。加强政府法制工作，健全政府法律顾问制度。优化执法体制，规范执法行为，加强执法监督。坚持问政于民、问计于民、问需于民，认真听取和采纳各民主党派和社会各界意见建议，推动网络问政、电视问政和网上建言献策。完善决策机制和程序，建立决策问责和纠错制度。加强政务微博、微信平台建设和政务、村务公开。

　　改进工作作风。贯彻落实中央“八项规定”，以开展党的群众路线教育实践活动为契机，着力强化政府作风建设，深入整治“四风”和“庸懒散拖”等行为。加强机关效能建设，强化市政府领导的领导责任、专项工作牵头部门的具体责任，鼓励创新、宽容失误、激励担当。严控各类考核检查、评比表彰活动，进一步精简会议和文件，实现三分之一以上市政府会议以电视电话会议方式召开。督促广大干部求真务实抓落实，深入基层解民忧。推动政府管理重心下移，加强乡镇（街道）建设，减轻基层负担。开展重点窗口单位和重点民生领域治理行动。强化督查督办，严格行政问责，确保政令畅通。

　　加强廉政建设。加强廉政风险防控，建立投资项目咨询论证制度，完善“限额设计”操作程序，严把政府投资项目立项关、概算关和建设关。实现公共资源交易“管办分离”，推进省市县三级平台互联互通。健全工程建设和政府采购网上招标、资格后审机制。加强经济责任审计和绩效审计，强化对重点部门、关键岗位、敏感环节监管。加强投资评审、内部审计、行政监察工作，严肃查处各类违法违纪案件。实行全口径预算管理，探索开展预算执行绩效跟踪工作。规范国资管理和非税收入监管。严控“三公”经费开支，严禁楼堂馆所建设。

　　六、全面深化改革

　　坚持把改革创新贯穿于政府工作的各领域各环节，推动政府职能向创造良好环境、提供优质服务、维护公平正义转变，促进市场环境、创业条件和干部作风根本好转。以解决问题为导向，认真贯彻《中共韶关市委关于全面深化改革的实施意见》，着力攻坚克难，深化重点领域和关键环节改革，力争获得更大的改革红利。结合转变政府职能，突出经济体制改革这个重点，全面完成商事登记制度改革，抓实政府机构、行政审批、事业单位分类管理、投资管理体制、公共资源交易和招投标、政府融资和债务管理、财税体制、金融发展、国有企业、社会信用、土地制度、资源价格等方面的改革，充分发挥市场在资源配置中的决定性作用，更好发挥政府作用，争创韶关营商环境、创新驱动、对外开放和产业竞争的新优势。

　　韶关的发展有赖于强化人才支撑。落实“人才强市”战略，全面推进“创新人才引进培养工程”，营造公平竞争、有为有位的环境。建立完善“乡贤”、专业技术人才、社工人才、企业经营管理人才信息库。加强招才引智工作，探索柔性引才新方式。继续推进市直机关干部跨部门交流。加强公务员队伍和产业工人队伍建设。着力培养视野开阔、敢于创新的企业家队伍。

　　各位代表，宏伟蓝图要靠实干来达成。让我们在省委、省政府和市委的正确领导下，抢抓机遇，攻坚克难，为推动韶关科学发展而努力奋斗！
